# Peritonitis secondary to hemorrhagic fever with renal syndrome: a case report in GuangZhou China

**DOI:** 10.1186/s12879-020-4775-8

**Published:** 2020-01-13

**Authors:** Na Huang, Nan Liu, Jianhui Lu

**Affiliations:** Emergency Department, The First Affiliated Hospital of Guangzhou University of Traditional Chinese Medicine, GuangZhou, China

**Keywords:** Hantavirus, Peritonitis, Infection

## Abstract

**Background:**

Hantavirus infection is worldwide epidemic and can cause life-threatening consequences. With more and more cases reported in countries with atypical morbidity, it is necessarily urgent to know some atypical symptoms and signs of Hantavirus infection.

**Case presentation:**

Here we report the case of a 44-year old male with a complaint of fever and diffuse abdominal pain, initially suspiciously diagnosed with acute peritonitis. The patient was eventually diagnosed as hemorrhagic fever with renal syndrome and enhanced CT scan showed peritonitis. The clinical condition of the patient was relatively mild and he was recovered 9 days later.

**Conclusion:**

Peritonitis secondary to hemorrhagic fever with renal syndrome is rare in clinically practice. When confronted with atypical celialgia, it is important to make differential diagnosis of hantavirus infection.

## Background

Hantaviruses, mainly including four kinds of viruses, Hantaan virus (HTNV), Seoul virus (SEOV), Puumala virus, Dobrava virus, are single-stranded, enveloped RNA viruses of the Bunyaviridae family, transmitted by mouse through their blood, saliva, urine and feces. And two of these, HTNV and SEOV are the major causative agents of HFRS in China [1]. With global communication more and more convenient, the number of countries, including UK, France, Netherlands, Germany and many states in US, reporting human cases of hantavirus infection is still on the rise [2]. HTV causes two life-threatening clinical syndromes in human beings: hemorrhagic fever with renal syndrome (HFRS) in Eurasia, and Hantavirus cardiopulmonary syndrome in America [3]. Typical symptoms of HFRS (in Asia, esp. China) includes: flu-like symptoms as fever, headache, generalized muscle and low back pain and gastrointestinal symptoms like anorexia, nausea, vomiting and diarrhea, and most importantly acute kidney injury. Classic HFRS occurs in five phases: fever, hypotension, oliguria, polyuria, and convalescence and its main serological manifestations include: leukocytosis, atypical lymphocytes, thrombocytopenia, increased serum-creatinine. But atypical symptoms and signs are often difficult to diagnose, or even misdiagnosed. It has been reported that HFRS can be misdiagnosed as calculous cholecystitis, acute pancreatitis, cholangitis, appendicitis, gastrointestinal hemorrhage. Here we report a patient complaining about fever and diffuse abdominal pain, initially suspected as peritonitis, eventually serologically proved to be HFRS. To our best knowledge, this is the first case of peritonitis associated with Hantavirus infection.

### Case presentation

**Fig 1:**
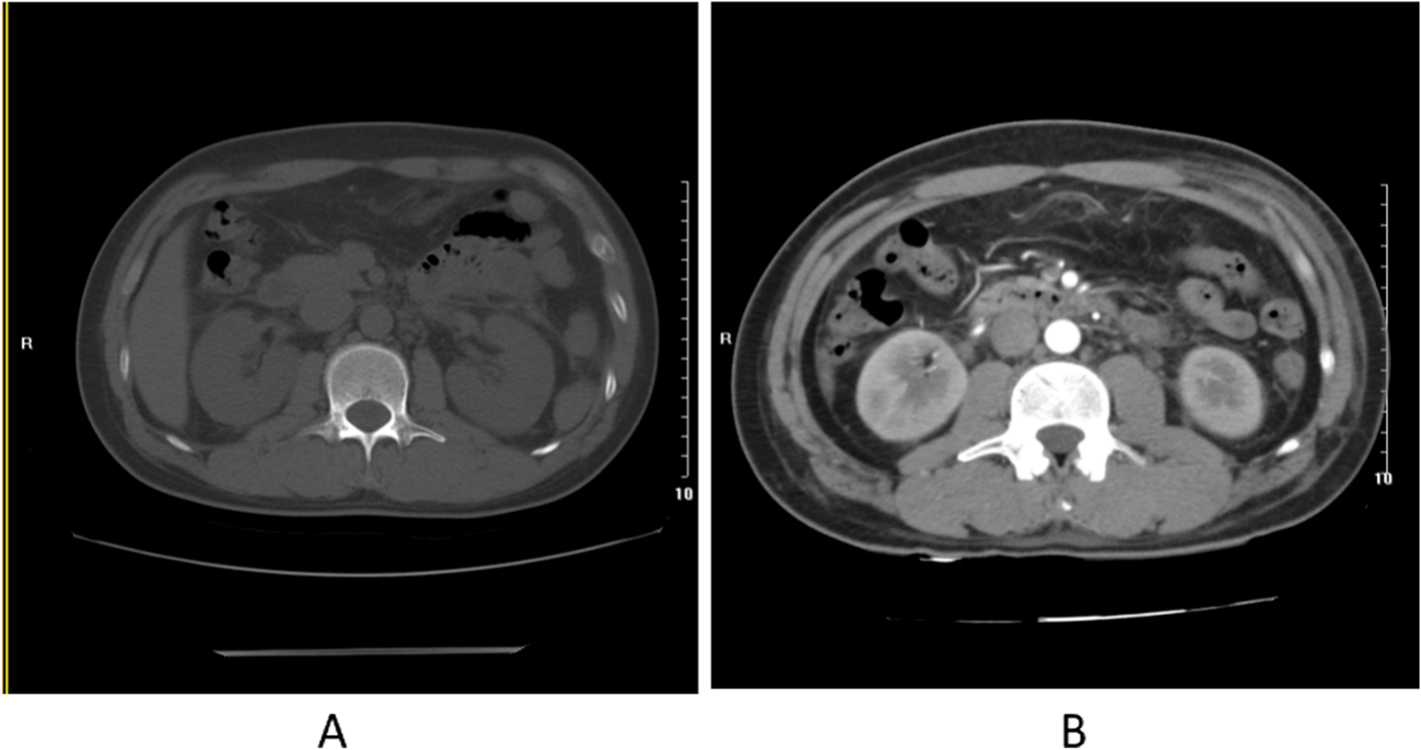
**a** was taken on May 14, 2019. **b** was taken on day May 19, 2019, which showed peritonitis, left abdominal wall swelling and edema, possible bilateral nephritis

In May 14, 2019, a 44-year-old man, presented with a 4-day history of fever, chills, headache, diffuse abdominal pain. He had no nausea, vomiting, or changes in bowel movements and denied other history of diseases. He lived in urban village in GuangZhou, China, where was overrun with rats. On admission he had a temperature of 40 °C and overwhelming diffuse abdominal stabbing pain. His blood pressure and heart rate were in the normal range and physical examination showed signs of peritoneal irritation positive, while Murphy’s sign and McBurney sign negative. His blood tests were as followed: white cell count 8.0 × 10^9^/L (reference range: 4–10), with 7.5 0 × 10^9^/L neutrophils (2–7.5), platelet count 253 × 10^9^/L (100–300), elevated creatinine 141 μmol/L (57–97), alanine aminotransferase 55 U/L (≤41), aspartate aminotransferase 52 U/L(≤40), lactic dehydrogenase 327 U/L (120–520), hydroxybutyrate 233 U/L (72–182), procalcitonin 1.61 ng/ml (0–0.05), D-dimers 608 ng/ml (0–324). Blood clotting and urinalysis were normal the whole time. Computerized tomography (CT) was carried out and the imaging investigations revealed minor thickening perirenal fascia, retroperitoneal multiple small lymph nodes, slight peritoneal and pelvic effusion (Fig. 1a). Based on these results and physical examination he was suspiciously diagnosed with acute peritonitis and treated with cefmetazole sodium, ornidazole, fluid replacement and supportive therapy for 4 days. His symptoms and blood indicators did not improve but progressed (Table 1). After consulting a chief surgeon, an explorative laparoscopy (May 18, 2019) was performed. The surgical intervention could not confirm the previous diagnose and found abdominal viscera and peritoneum normal. Three sets of blood culture for bacteria and fungus were negative. And another contrast-enhanced CT (May 19, 2019) (Fig. 1b) was performed and revealed peritonitis, left abdominal wall swelling and edema, possible bilateral nephritis, retroperitoneal multiple small enlarged lymph nodes, hepatomegaly. Serum taken 5 days after admission (May 19, 2019) showed hantavirus were positive for both IgG and IgM HTV-specific antibodies (immunofluorescence assay), with IgG antibody titer 1:568. Also, ascites from laparoscopy surgery showed IgM antibody positive and IgG antibody titer 1:320. Based on symptoms and signs, along with his complete blood count, creatinine level, urine volume changes, he was eventually diagnosed as HFRS (mild) and secondary peritonitis. Antibiotics and supportive therapy were applied. He was basically recovered and discharged on May 23, 2019.

**Table 1:** shows changes of the highest body temperature (Tmax), 24-h urine volume after admission and blood indicators

	2019/5/14	2019/5/15	2019/5/16	2019/5/17	2019/5/18	2019/5/19	2019/5/20	2019/5/21	2019/5/22	2019/5/23
WBC	8.3	18.6	–	15.86	17.4	15.3	15.1	–	–	15.1
NEU	6.6	12.5	–	12.9	14.8	12.5	11.3	–	–	12.3
LYM	3.91	3.8	–	1.65	1.42	1.42	2.2	–	–	2.02
HCT	0.372	0.372	–	0.331	0.348	0.333	0.354	–	–	0.389
PLT	141	150	–	147	183	183	221	–	–	278
CREA	141	191	–	242	266	175	130	–	–	108
Ur	6.67	6.26	–	9.28	8.53	8.48	9.82	–	–	5.04
Tmax	36.8	37.4	38	37.8	37.4	36.8	37	36.6	36.5	36.3
24 h-urine	–	980	2090	3400	3260	4010	3350	2350	3050	3100

*WBC* White blood cell, *NEU* Neutrophils, *LYM* Lymphocyte, *HCT* Hematocrit, *Ur* Urea, *PLT* Platelet count, *CREA* Creatinine. WBC, NEU, LYM, PLT measured by × 109/L (reference range: 4–10, 2–7.5, 1.6–4.0), HCT (0.40–0.50), Ur measured by mmol/L (3.1–8.0), CREA measured by μmol/L (57–97), Tmax measured by Celsius degree, urine volume measured by milliliter

## Discussion and conclusion

FRS can be manifested as various symptoms due to its multi-system injury. However, a hallmark of the disease is its acute onset with chills, fever and malaise, and abdominal pain; patients typically have thrombocytopenia and elevation of serum creatinine level [4]. Serum specific antibody or viral RNA helps make a definite diagnose. Our patient did not show any signs of thrombocytopenia, and we were not able to perform RT-PCR to make a molecular test because of condition limitations. But his symptoms and signs, creatinine level, urine volume changes, all indicated HFRS. Most importantly, his blood and ascites test verified the diagnosis.

Celialgia is a common symptom among many medical and surgical diseases, and inflammation of all internal organs can cause abdominal pain. It was revealed that the course of HFRS was similar to acute abdomen in 13.2% of acute cholecystitis, acute pancreatitis, and acute appendicitis [5]. With peritoneal irritation positive, abdomen pain with fever can be easily misdiagnosed. If the abdominal CT scan shows no focus, However, we should also pay close attention to changes in creatinine level, and 24-h urine output. With elevated creatinine, oliguria or polyuria, tests for hantavirus should be carried out.

In conclusion, it is important that a differential diagnosis of hantavirus infection should be considered, when confronted with celialgia, especially if results show multiple organs injury and urine volume change. In addition, history is also very important, potential exposure to rodents or their excretions may suggest hantavirus infection.

## Data Availability

The data set of this article will not be available publicly, to ensure the patient’s privacy, but are available from the corresponding author on reasonable request.

## Abbreviations

CREA: Creatinine
CT: Computerized tomography
HCT: Hematocrit
HFRS: Hemorrhagic fever with renal syndrome
HTV: Hantavirus
LYM: Lymphocyte
NEU: Neutrophils
PLT: Platelet count
Ur: Urea
WBC: White blood cell
